# Supplementary material for: In-Silico Functional Metabolic Pathways Associated to Chlamydia trachomatis Genital Infection
Source: Int J Mol Sci. 2022 Dec 13;23(24):15847. doi: 10.3390/ijms232415847 (PMC9781786; doi:10.3390/ijms232415847)
Supplement: Supplementary file 1 [file ijms-23-15847-s001.zip › Figure_S1.pdf]

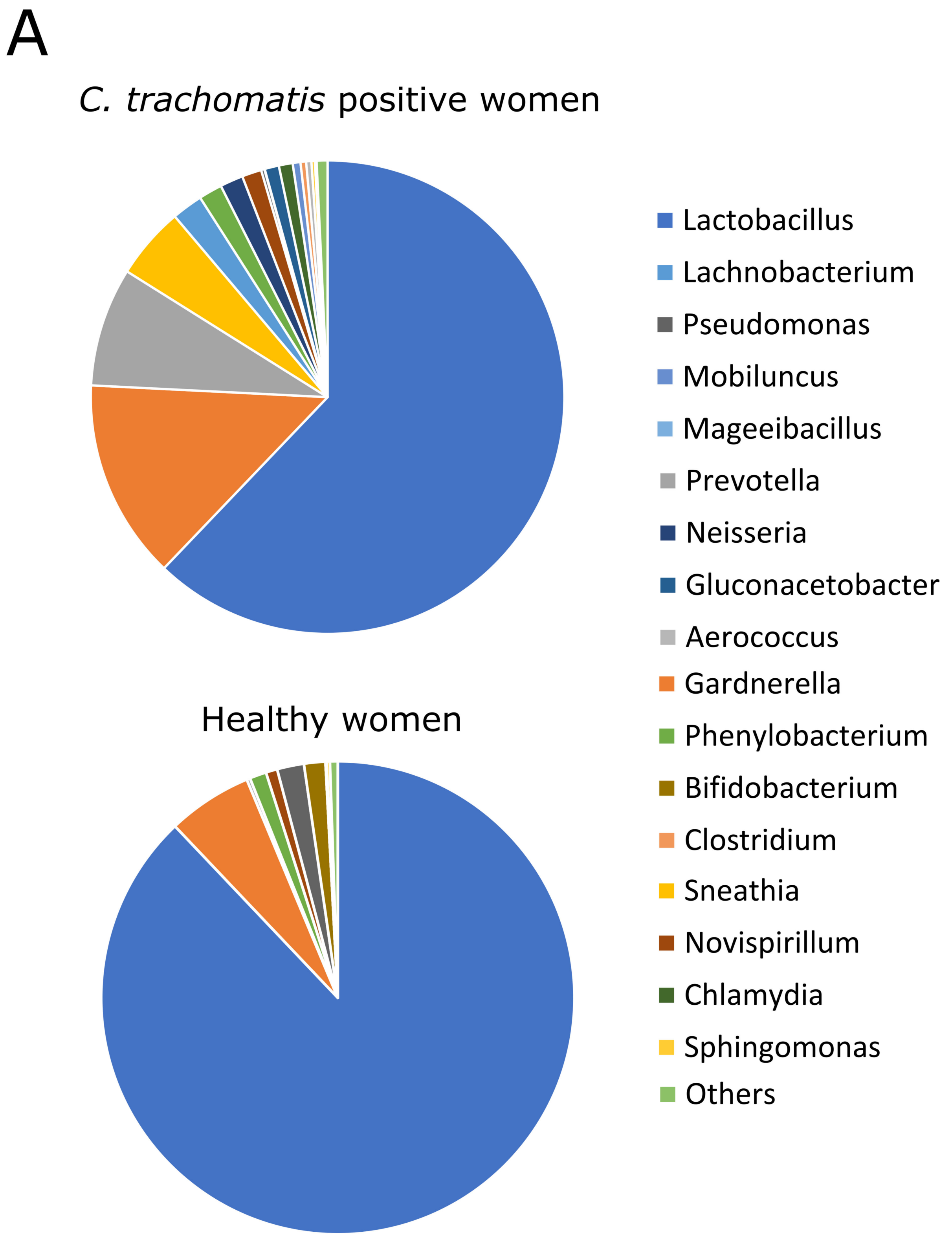

B

Community state type	% of women (no. of women [*n*]) *C. trachomatis*-positive women (*n*=39)	Healthy women (*n*=99)	*p* values
CST-I (*L. crispatus*)	25.64 (10)	56.57 (56)	0.001
CST-II (*L. gasseri*)	0.00 (0)	17.17 (17)	0.003
CST-III (*L. iners*)	35.89 (14)	20.20 (20)	N.S.
CST-IV (anaerobic bacteria)	38.46 (15)	6.06 (6)	0.000002

CST, Community State Type

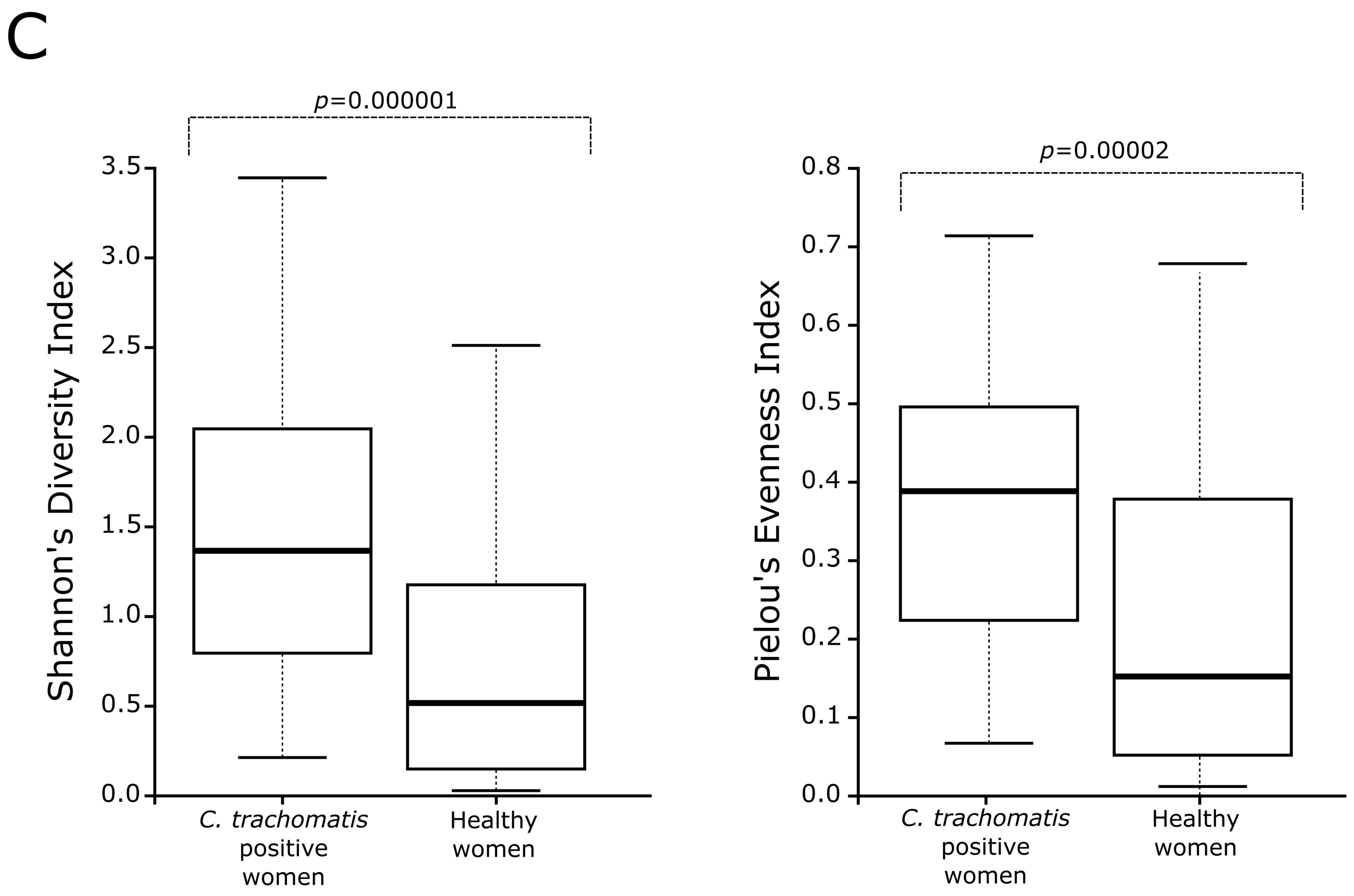

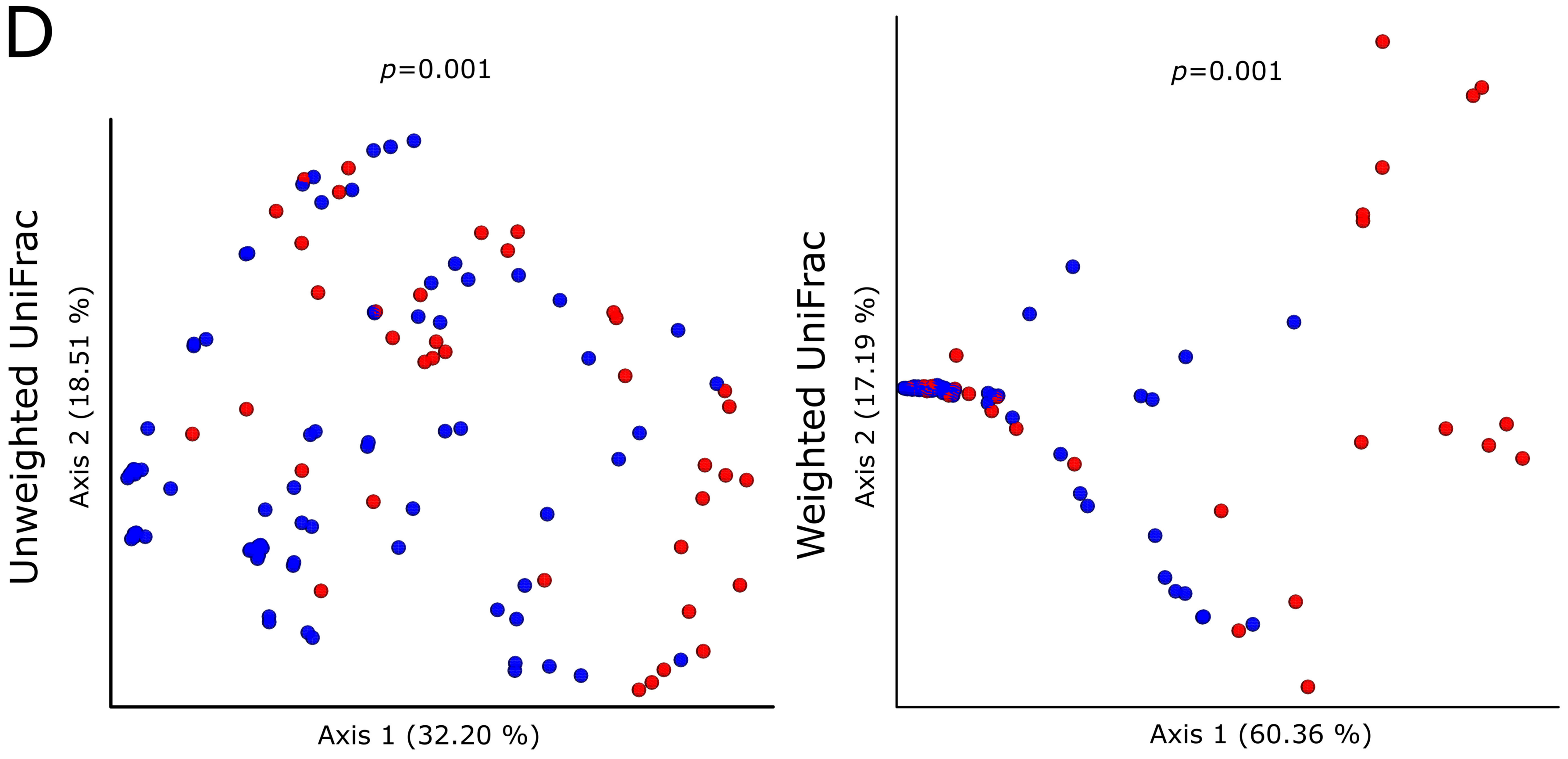

Figure S1. Cervicovaginal microbiota characteristics in *C. trachomatis*-positive women and healthy controls. (**A**) Schematic composition at the genus level of the main bacterial species; (**B**) classification of the cervicovaginal microbiota of *C. trachomatis*-positive women and healthy controls in the respective CSTs; (**C**) Shannon's and Pielou's evenness indexes as measures of alpha-diversity within groups; (**D**) Principal coordinate analysis of Unweighted and weighted UniFrac as measures of beta diversity between groups. Each circle represents the cervicovaginal microbiota of each woman.
